# Vaccine-Induced Subcutaneous Granulomas in Goats Reflect Differences in Host–Mycobacterium Interactions between BCG- and Recombinant BCG-Derivative Vaccines

**DOI:** 10.3390/ijms231910992

**Published:** 2022-09-20

**Authors:** Elisabeth M. Liebler-Tenorio, Johannes Heyl, Nadine Wedlich, Julia Figl, Heike Köhler, Gopinath Krishnamoorthy, Natalie E. Nieuwenhuizen, Leander Grode, Stefan H. E. Kaufmann, Christian Menge

**Affiliations:** 1 Institute of Molecular Pathogenesis, Friedrich-Loeffler-Institut, 07743 Jena, Germany; 2 Department of Immunology, Max Planck Institute for Infection Biology, 10117 Berlin, Germany; 3 Vakzine Projekt Management GmbH, 30625 Hannover, Germany; 4 Max Planck Institute for Multidisciplinary Sciences, 37077 Göttingen, Germany; 5 Hagler Institute for Advanced Study, Texas A&M University, College Station, TX 77843, USA

**Keywords:** tuberculosis, vaccination, recombinant BCG, goat, histology, immunohistochemistry, lymphocytes, foam cells, multinucleated giant cells

## Abstract

Tuberculous granulomas are highly dynamic structures reflecting the complex host–mycobacterium interactions. The objective of this study was to compare granuloma development at the site of vaccination with BCG and its recombinant derivatives in goats. To characterize the host response, epithelioid cells, multinucleated giant cells (MNGC), T cell subsets, B cells, plasma cells, dendritic cells and mycobacterial antigen were labelled by immunohistochemistry, and lipids and acid-fast bacteria (AFB) were labelled by specific staining. Granulomas with central caseous necrosis developed at the injection site of most goats though lesion size and extent of necrosis differed between vaccine strains. CD4^+^ T and B cells were more scarce and CD8^+^ cells were more numerous in granulomas induced by recombinant derivatives compared to their parental BCG strain. Further, the numbers of MNGCs and cells with lipid bodies were markedly lower in groups administered with recombinant BCG strains. Microscopic detection of AFB and mycobacterial antigen was rather frequent in the area of central necrosis, however, the isolation of bacteria in culture was rarely successful. In summary, BCG and its recombinant derivatives induced reproducibly subcutaneous caseous granulomas in goats that can be easily monitored and surgically removed for further studies. The granulomas reflected the genetic modifications of the recombinant BCG-derivatives and are therefore suitable models to compare reactions to different mycobacteria or TB vaccines.

## 1. Introduction

Tuberculosis is a disease caused by mycobacteria belonging to the Mycobacterium Tuberculosis Complex (MTC). *Mycobacterium tuberculosis*, a member of MTC, continues to cause high morbidity and mortality in humans [1,2], and *Mycobacterium (M.) bovis* is an important pathogen in many animal species and a zoonotic agent for humans [3,4,5]. Vaccines are an essential preventive measure in the fight against tuberculosis. New vaccines should provide better protection and/or a better safety profile compared to the widely used Bacillus Calmette-Guérin (BCG), an attenuated live vaccine based on *M. bovis* which was developed more than 100 years ago [6,7]. Before vaccine candidates enter clinical trials in humans, immunogenicity, safety and protection have to be evaluated [8]. These evaluations may include the investigation of granulomas in model systems as surrogates for mycobacterial infections [9,10,11,12,13,14].

Granulomas are characteristic lesions of tuberculosis (TB) developed during the interaction between host cells and mycobacteria to prevent disease progression. At the same time, these cellular aggregates can provide a niche for the pathogen`s survival [4,15,16]. Studying granuloma progression and formation in experimental animal TB models including rodents, guinea pig, ruminants and non-human primates allows for insights into these complex processes [17]. Yet, it is difficult, as experimental TB infection studies have to be strictly performed at biosafety level 3 containment facilities. Granulomas may also develop locally at the application site of BCG, which can be studied at lower biosafety levels. BCG-induced granulomas have been reported as uncommon adverse reactions, especially after subcutaneous vaccination in humans [18,19,20,21]. In contrast, the subcutaneous injection of BCG or other mycobacteria-based vaccines frequently induces granulomas at the vaccination site in ruminants [22,23,24,25,26]. 

Goats vaccinated subcutaneously with BCG vaccine or its recombinant Δ*ureC::hly* (VPM1002) derivatives invariably developed granulomas at the injection sites [27]. The recombinant BCG vaccine VPM1002 was designed to modify the in vivo behavior of BCG in infected antigen-presenting cells [28,29,30,31]. To this end, the gene for listeriolysin O (LLO) of *Listeria monocytogenes* was inserted, and the gene encoding urease C was deleted from the genome of BCG. The pore-forming protein LLO perturbates the membrane of phagosomes containing BCG and allows mycobacterial antigens, enzymes and DNA to translocate into the cytosol, which ultimately results in profound changes in the intracellular processing, including apoptosis and autophagy pathways, inflammasome activation as well as antigen presentation via MHC I to CD8^+^ T cells, and thus improves the activation of the immune system [31,32,33]. The additional deletion of the urease C-encoding gene prevents neutralization of the acidic pH in phagosomes, which is required for optimal LLO bioactivity. The better protection and safety of VPM1002 compared to BCG were revealed in challenge experiments in mice [28,34,35,36]. Phase I and phase II vaccine trials in humans have shown an improved safety profile for VPM1002 compared to BCG [37,38,39]. The VPM1002 derivative BCG Δ*ureC::hly* Δ*pdx1* (PDX) has an additional deletion which results in auxotrophy for vitamin B6 and further increases the safety in immunocompromised hosts [40]. The apoptotic death of infected cells is considered beneficial for the induction of immune responses in the host [32]. Hence, the anti-apoptotic *nuoG* gene was deleted in VPM1002, and the derivative BCG Δ*ureC::hly* Δ*nuoG* (NUOG) conferred even better protection than that induced by VPM1002 in mice [41]. 

In this study, subcutaneous granulomas induced by BCG, the recombinant BCG candidate vaccine VPM1002 and two modifications of VPM1002 were examined in goats. Goats have been described as developing caseous granulomas in response to mycobacterial infections, e.g., *M. bovis*, *M. caprae*, *M. tuberculosis*, *M. avium* subsp, *paratuberculosis* and *M. avium* subsp, *hominissuis* [4,42,43,44,45,46]. The cellular composition and organization of granulomas were characterized and correlated with the in vivo behavior of the BCG vaccine and its recombinant derivatives to determine whether vaccine-induced granulomas in goats could serve as models for the interaction between mycobacteria and their host. 

## 2. Results

### 2.1. Macroscopic Findings

**Figure 1:**
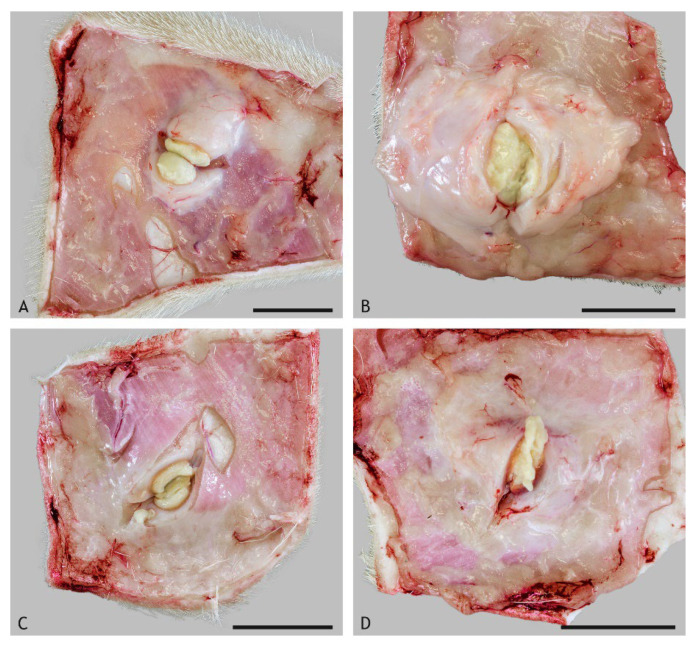
Gross morphology of caseous granulomas induced by the subcutaneous injection of BCG ((**A**), goat 28), VPM1002 ((**B**), goat 9), PDX ((**C**), goat 18) and NUOG ((**D**), goat 26) after incision. The necrotic material was firm, resulting in a smooth cut-surface in BCG- and PDX-induced granulomas and a soft, bulging out cut-surface in VPM1002- and NUOG-induced granulomas. Scale bars = 2 cm.

**Figure 2:**
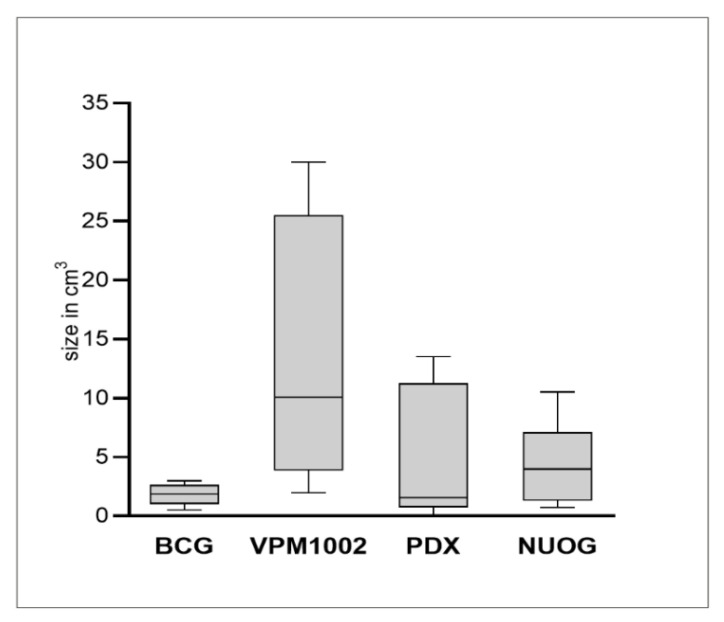
Size (volume) of granulomas induced in goats by subcutaneous injection of BCG, VPM1002, PDX and NUOG. Each column represents measurements from six goats. Interquartile range (grey box), median (line) as well as maximum and minimum size (whiskers) are indicated.

Granulomas with central caseous necrosis were present at the injection site in all goats vaccinated with BCG, VPM1002 and NUOG and in four out of six goats vaccinated with PDX, but they were present in none of the control-treated goats. Granulomas varied in size, with the smallest seen after vaccination with BCG and the largest seen after vaccination with VPM1002 (Figure 1 and Figure 2). Size differences were not statistically significant because of the small number of animals and the marked variation between individual goats. BCG- and PDX-induced granulomas had firm, solid necrotic centers, while VPM1002- and NUOG-induced granulomas were filled with liquified, creamy material containing calcified granules (Figure 1). 

### 2.2. Morphologic Characteristics and Organization of Granulomas

**Figure 3:**
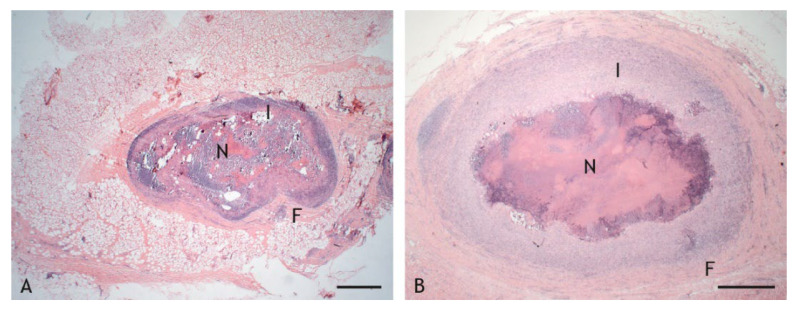
Granulomas with central necrosis (N) surrounded by an inflammatory infiltrate (I) of variable width and enclosed by a fibrous connective tissue capsule (F). (**A**). BCG-induced granuloma, goat 15: thin inflammatory infiltrate and thin connective tissue capsule. (**B**). VPM1002-induced granuloma, goat 1: wide inflammatory infiltrate and wide connective tissue capsule. Hemalaun-eosin (HE) stain. Scale bars = 100 µm.

Caseous granulomas were characterized by central necrosis with a variable degree of calcification and a variable abundance of nuclear debris. Necrotic areas were surrounded by inflammatory cell infiltrates of variable width and cellular composition (Figure 3A,B). Granulomas were enclosed by fibrous connective tissue. They were classified as type 3 granulomas based on the classification scheme for tuberculous granulomas in ruminants by Wangoo et al. [47], with the exception of the lesions in two goats vaccinated with PDX. One of these had an accumulation of epithelioid cells and macrophages interspersed with a few lymphocytes and minimal necrosis, and the other goat had only a scar of connective tissue at the inoculation site. The lesions of these two goats were not included in further evaluations.

**Figure 4:**
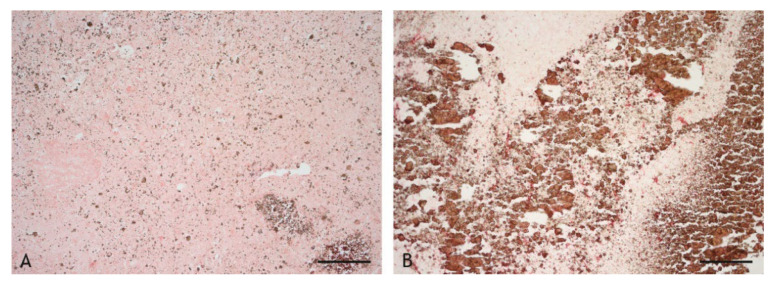
Different calcification patterns within the caseous necrosis in granulomas of goats after injection of BCG and VPM1002. Calcium precipitates are dark brown to black. (**A**). BCG-induced granuloma, goat 22: dusty to finely granular calcium precipitates. (**B**). VPM1002-induced granuloma, goat 9: coarse calcium precipitates. Von Kossa stain. Scale bars = 100 µm.

Mineralization due to calcium ion deposits, identified by von Kossa staining, was always present in the caseous necrosis (Table 1). Calcification was most extensive in NUOG-induced granulomas, moderate to severe in BCG- and VPM1002-induced granulomas and sporadic in PDX-induced granulomas. Mostly granular to dusty precipitates mixed with coarse calcium deposits were observed (Figure 4). In VPM1002-induced granulomas, coarse calcium precipitates were predominantly presented in four out of six goats. 

**Figure 5:**
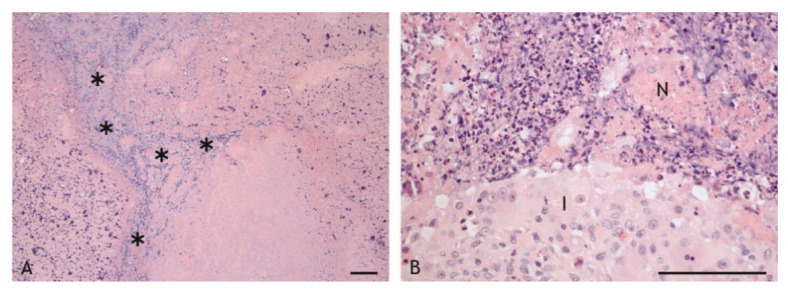
Nuclear debris (**A**) and neutrophils (**B**) in the caseous necrosis of a VPM1002-induced granuloma, goat 1. (**A**). Nuclear debris (*) extends in a wavy pattern throughout the necrosis. (**B**). Groups of neutrophils are multifocally present at the transition zone between inflammatory infiltrate (I) and necrosis (N). HE stain. Scale bars = 100 µm.

Nuclear debris was another common feature noted in the caseous necrosis. It accumulated at the outer edge of the necrosis and extended in a wavy fashion throughout the necrosis with no uniform distribution (Figure 5A). Groups of intact and degenerated neutrophils were present at the transition zone between necrosis and inflammatory infiltrate extending into the necrosis. They were particularly numerous in VPM1002-induced granulomas (Figure 5B).

**Figure 6:**
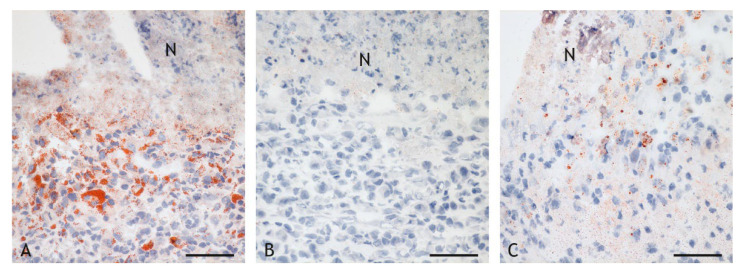
Lipid droplets in foam cells of caseous granulomas. (**A**). BCG-induced granuloma, goat 22: Many epithelioid cells and MNGCs adjacent to the necrosis (N) contain numerous lipid droplets (red). Lipid droplets are also present in the necrosis. (**B**). VPM1002-induced granuloma (goat 9): There are no lipid droplets present adjacent to or in the necrosis. (**C**). NUOG-induced granuloma (goat 20): A few large, pleomorphic lipid deposits are present in a few epithelioid cells adjacent to the necrosis. Sudan III stain. Scale bars = 50 µm.

The inflammatory cells bordering the necrosis were predominantly epithelioid cells, macrophages and, occasionally, multinucleated giant cells (MNGC) in different degrees of disintegration. These cells had a vacuolated cytoplasm, as described for foam cells, especially in the BCG-induced granulomas. Staining with Sudan III revealed numerous small intracytoplasmic lipid droplets in many epithelioid cells and some MNGCs (Table 2, Figure 6A). Free lipid droplets were present within the necrosis but were seldom present in VPM1002- and PDX-induced granulomas (Figure 6B), whereas moderate numbers of epithelioid cells with lipid droplets of variable sizes and numbers were present throughout the inflammatory infiltrate in NUOG-induced granulomas (Figure 6C).

**Figure 7:**
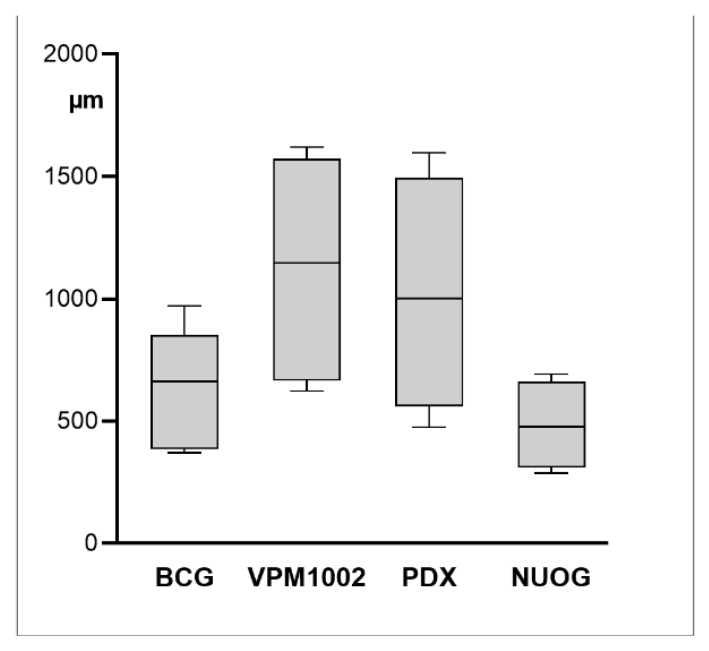
Width of inflammatory cell infiltrate of granulomas in goats after subcutaneous injection of BCG, VPM1002, PDX or NUOG. Width was measured in HE-stained paraffin sections at eight equidistant sites per granuloma. Each column represents measurements from six goats. Interquartile range (grey box), median (line) as well as maximum and minimum width (whiskers) are indicated.

**Figure 8:**
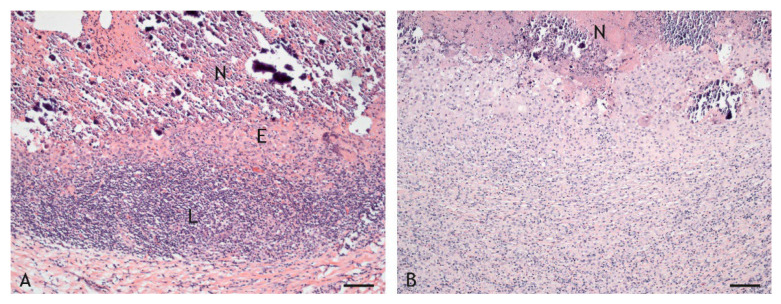
Differences in width and thickness of inflammatory infiltrate. (**A**). BCG-induced granuloma, goat 15: distinct area of epithelioid cells (E) adjacent to the necrosis (N) followed by lymphocytes (L). (**B**). VPM1002-induced granuloma, goat 1: epithelioid cells and lymphocytes are mixed throughout the infiltrate adjacent to the necrosis (N). HE stain. Scale bars = 100 µm.

The inflammatory cell infiltrates were of variable width (Figure 3 and Figure 7). Given the wide variation between individuals, and even within granulomas, such variation was not statistically significant (*p* ≤ 0.05). Overall, however, the layer of cells was relatively wider in VPM1002- and PDX-induced granulomas and thinner in BCG- and NUOG-induced granulomas. The inflammatory infiltrates also varied in their organization. Some granulomas had a stratified organization with a distinct central shell of predominantly epithelioid cells and a peripheral shell of predominantly lymphocytes (Figure 8A). In others, all cell types were evenly mixed (Figure 8B). The pattern of organization changed even within granulomas. Regardless, there was no clear association with any of the vaccines.

**Figure 9:**
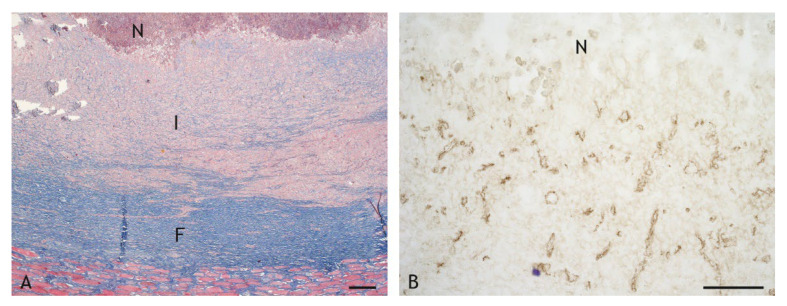
Fibrous connective tissue capsule (**A**) and vascularization (**B**) in VPM1002-induced granulomas (N–central necrosis). (**A**). Fibroblasts and collagen fibers (blue) form a thick capsule (F) around the inflammatory cell infiltrate (I). The circular orientation allows for discrimination from preexisting subcutaneous connective tissue (goat 1). Azan stain. Scale bar = 200 µm. (**B**). Many small blood vessels permeate throughout the inflammatory infiltrate even adjacent to the necrosis (N, goat 9). Immunohistochemistry (IHC), von Willebrand Factor. Scale bar = 100 µm.

Granulomas were enclosed by fibroblasts and collagen fibers predominantly in a circular orientation (Figure 9A). Radial orientation was occasionally seen in VPM1002- and PDX-induced granulomas. Inflammatory cell infiltrates were frequently observed within the connective tissue capsule. Small blood vessels were present in moderate-to-high numbers throughout the inflammatory infiltrate, even close to the necrosis (Figure 9B). Perivascular infiltrates of inflammatory cells were especially frequent in the subcutaneous connective tissue surrounding NUOG-induced granulomas. 

### 2.3. Cell Types in the Inflammatory Infiltrate

**Figure 10:**
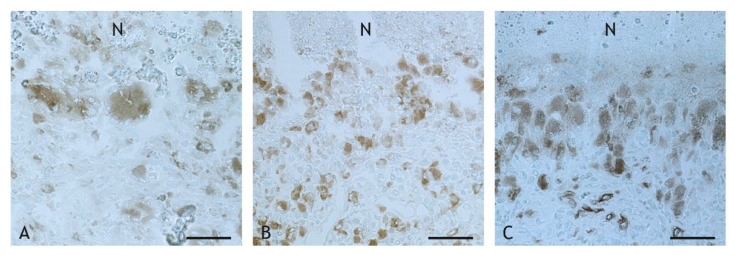
Epithelioid cells and MNGCs adjacent to the central necrosis (N). (**A**). BCG-induced granuloma, goat 7: epithelioid cells and MNGCs. (**B**). VPM1002-induced granuloma, goat 24: predominantly epithelioid cells. (**C**). NUOG-induced granuloma, goat 5: predominantly very large epithelioid cells bordering the necrosis. IHC, CD68. Scale bars = 50 µm.

**Figure 11:**
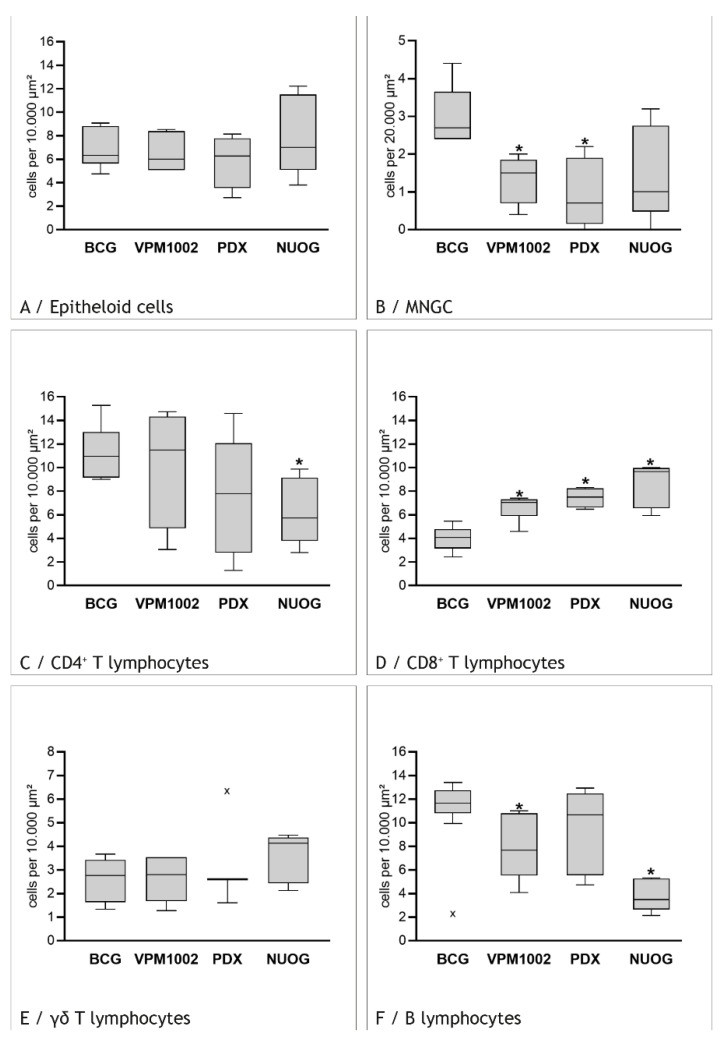
Composition of inflammatory cell infiltrates in BCG-, VPM1002-, PDX- and NUOG-induced granulomas. Each column represents 6 goats, with 15 reference areas counted per goat. Interquartile range (IQR, grey box), median (line) as well as maximum and minimum (whiskers) are indicated. x outliers, more than 1.5 IQR below first quartile or above third quartile, * significant differences (*p* < 0.05) from BCG-induced granulomas.

Epithelioid cells were numerous in all granulomas but without any significant differences between vaccination groups (Figure 10 and Figure 11A). Overall, they were preferentially localized around the necrosis (Figure 10A–C). In VPM1002-induced granulomas, they were present throughout the infiltrate but had smaller sizes in the periphery. The highest numbers of epithelioid cells were detected in NUOG-induced granulomas. These epithelioid cells were particularly large (30–40 µm in diameter), with extensive cytoplasm often containing nuclear fragments. Some of these cells appeared to be fused but did not display MNGC morphology, which is characterized by many nuclei at the periphery or the center.

MNGC were present in markedly lower numbers than all other cell types and were detected only close to the necrosis. Therefore, their number was evaluated in a larger area (2 × 10^4^ µm^2^) and only next to the necrosis. The highest number of MNGCs with Langhans cell morphology was consistently detected in BCG-induced granulomas (Figure 10A). Their number was lower in granulomas induced by VPM1002 (Figure 10B) or its derivatives, with significant (*p* ≤ 0.05) differences for VPM1002 and PDX compared to BCG (Figure 11B). In addition, MNGCs were smaller in VPM1002- and PDX-induced granulomas compared to BCG-induced granulomas.

**Figure 12:**
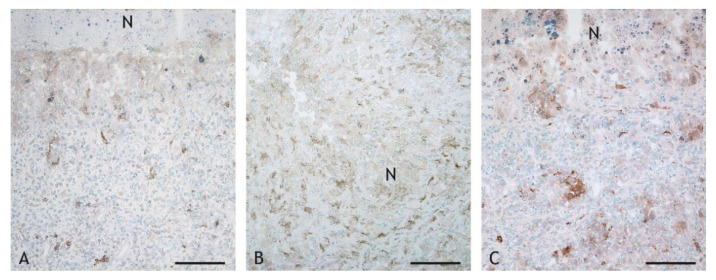
MHC II^+^ DCs adjacent to the central necrosis (N). (**A**). VPM1002-induced granuloma, goat 24: moderate numbers of DCs associated with epithelioid cells. (**B**). PDX-induced granuloma, goat 19: dense network of DCs adjacent to the necrosis. (**C**). NUOG-induced granuloma, goat 5: prominent DCs in close contact with epithelioid cells. IHC, MHC II. Scale bars = 100 µm.

Dendritic cells (DCs) were identified based on morphology and the intense expression of MHC II on cytoplasmic extensions. DCs were present in the inflammatory infiltrate adjacent to the necrosis, predominantly in close association with epithelioid cells and MNGCs. Moderate numbers were seen in BCG- and VPM1002- induced granulomas (Figure 12A), while a dense network was seen in PDX-induced granulomas (Figure 12B). In NUOG-induced granulomas, the intense labelling of DCs was observed in proximity of the large epithelioid cells (Figure 12C). 

**Figure 13:**
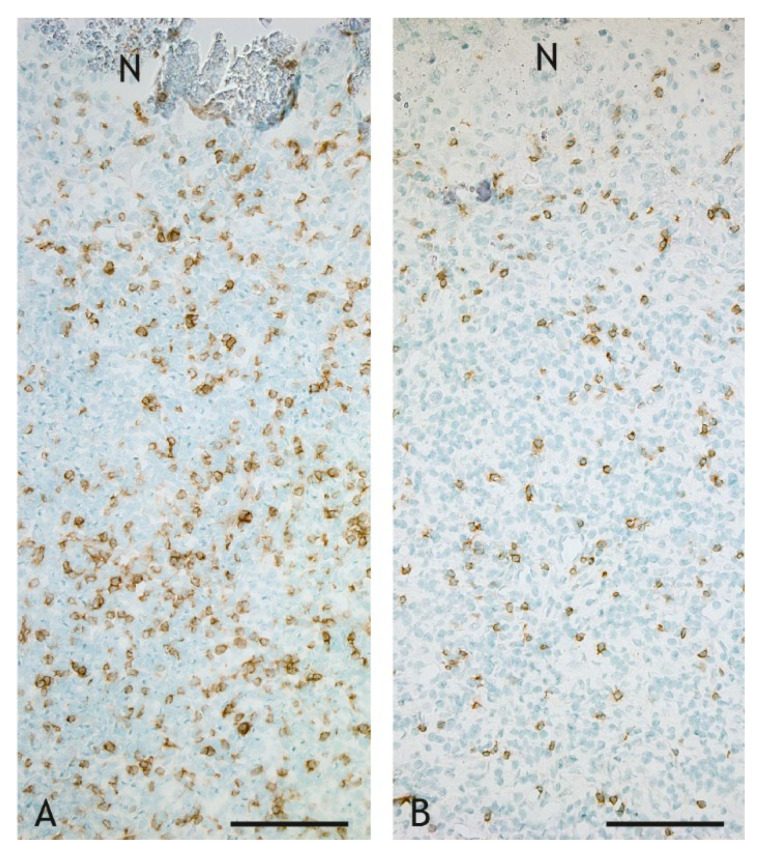
CD4^+^ T cells in the inflammatory infiltrate of granulomas. The number of CD4^+^ T cells is higher in the BCG-induced granuloma ((**A**), goat 13) compared to the VPM1002-induced granuloma ((**B**), goat 24). IHC, CD4. Scale bars = 100 µm.

CD4^+^ T cells were the lymphocyte subtype most frequently detected in the inflammatory infiltrate, but they also had the highest individual variation (Figure 11C and Figure 13A,B). The number of CD4^+^ T cells was lower in VPM1002-, PDX- and NUOG-induced granulomas compared to BCG-induced granulomas, with significant (*p* ≤ 0.05) differences between BCG and NUOG. While CD4^+^ T cells were evenly distributed throughout the inflammatory infiltrate in PDX- and NUOG-induced granulomas, there was a gradient in BCG- and VPM1002-induced granulomas, with lower numbers of CD4^+^ T cells towards the central necrosis. 

**Figure 14:**
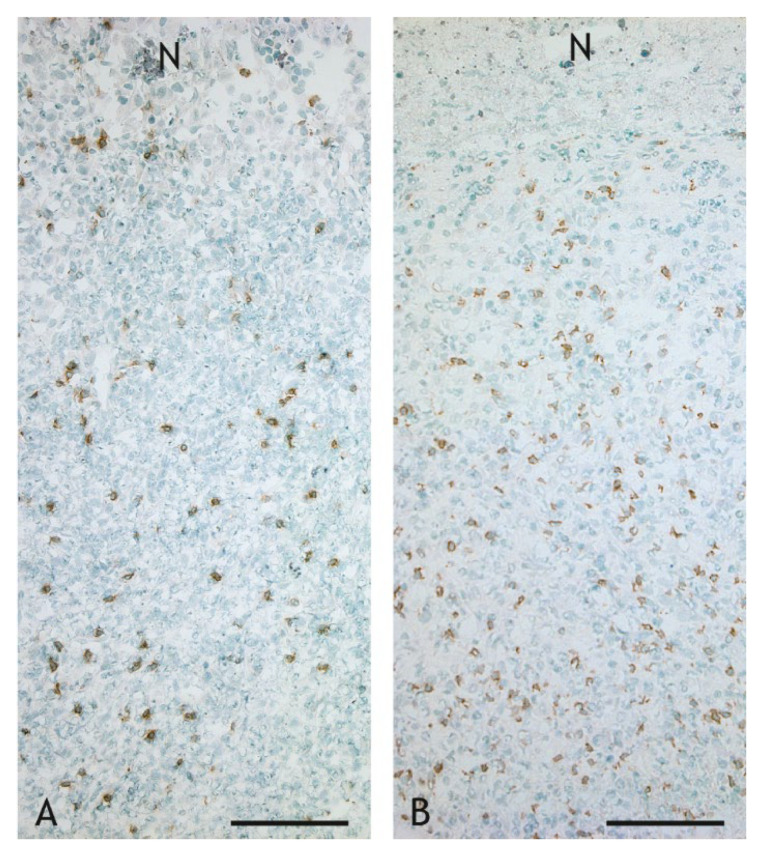
CD8^+^ T cells in the inflammatory infiltrate of granulomas. The number of CD8^+^ T cells is higher in the VPM1002-induced granuloma ((**B**), goat 24) compared to the BCG-induced granuloma ((**A**), goat 13). IHC, CD8. Scale bars =100 µm.

CD8^+^ T cells were detected with lower frequency than CD4^+^ T cells in the inflammatory infiltrate (Figure 11D and Figure 14A,B). Compared to BCG-induced granulomas, where the ratio of CD4^+^:CD8^+^ T cells was 3:1, their number was significantly (*p* ≤ 0.05) increased in VPM1002-, PDX- and NUOG-induced granulomas, resulting in CD4^+^:CD8^+^ T cell ratios of 1.4:1, 1:1 and 0.6:1, respectively. The distribution of CD8^+^ T cells in the inflammatory infiltrate was comparable to that of CD4^+^ T cells. 

γδ T cells were the lymphocytes present in the lowest numbers and were evenly distributed throughout the inflammatory infiltrate. There were no differences between the vaccine groups (Figure 11E). 

**Figure 15:**
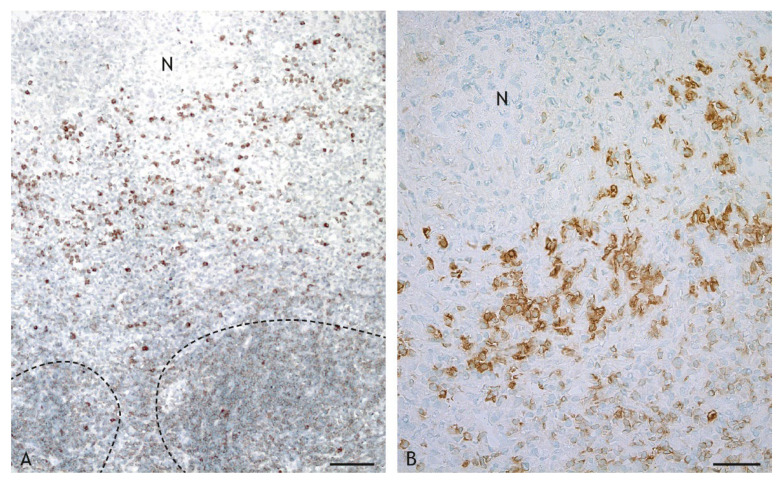
B cells and plasma cells in the inflammatory infiltrate of granulomas with central necrosis (N). (**A**). BCG-induced granuloma, goat 13: B cells (weak labelling) form follicle-like structures in the periphery of the inflammatory infiltrate (indicated by a hatched lines). (**B**). PDX-induced granuloma, goat 19: groups of plasma cells (intensely labelled cytoplasm) are present adjacent to the central necrosis (N). IHC, CD79α. Scale bar in (**A**) = 100 µm, scale bar in (**B**) = 50 µm.

B cells were present in similar numbers as CD4^+^ T cells (Figure 11F). The number of B cells was lower in VPM1002-, PDX- and NUOG-induced granulomas compared to BCG-induced granulomas. B cells were found throughout the inflammatory infiltrate but were more numerous in the periphery. They formed multiple follicle-like structures in the periphery of BCG-, VPM1002- and PDX-induced granulomas (Figure 15A). Plasma cells were present in lower numbers compared to B cells throughout the inflammatory infiltrate (Figure 11F). There were no differences in the overall numbers between the different vaccine types, but clusters of plasma cells adjacent to the necrosis were particularly prominent in VPM1002- and PDX-induced granulomas (Figure 15B). 

### 2.4. Mycobacteria in the Granuloma

**Figure 16:**
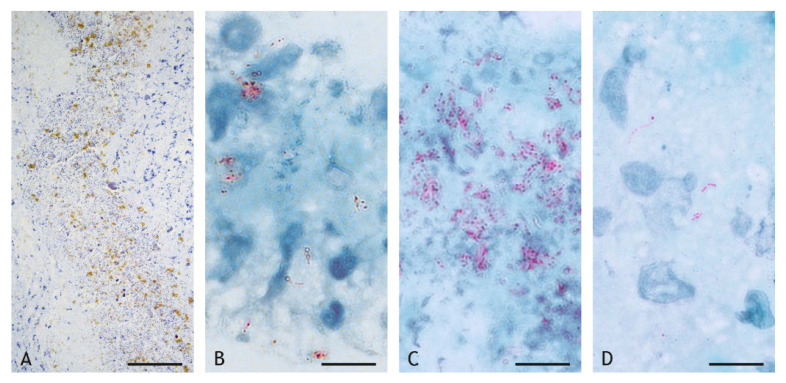
Mycobacterial antigen (brown) and acid-fast bacilli (AFB, red) in the central necrosis (**A**–**C**) and in the inflammatory infiltrate (**D**). (**A**). Patchy distribution of mycobacterial antigens (brown) in the central necrosis (goat 20, NOUG). (**B**,**C**): Number of AFB (red) in the central necrosis varies markedly between individuals (B, goat 13, BCG; C, goat 20, NUOG). (**D**). Few AFB (red) are present in epithelioid cells of the inflammatory infiltrate (D, goat 20, NUOG). (**A**). IHC, mycobacterial antigen, scale bar = 100 µm; (**B**–**D**). Ziehl–Neelsen (ZN) stain, scale bars = 10 µm.

Acid-fast bacilli (AFB) and mycobacterial antigens were frequently detected in particularly high abundance within the caseous necrosis (Figure 16A–D, Table 3). The distribution was not even but often focal, associated with areas of cellular debris and calcification. In the inflammatory infiltrate, AFB were only found occasionally in low numbers, free or within epithelioid cells (Figure 16D). Mycobacteria were detected in all BCG- and VPM1002-induced granulomas, in three of four PDX-induced granulomas and in five of six NUOG-induced granulomas. 

Cultural isolation was successful in three BCG-, two VPM1002- and one NUOG-induced granulomas, but it was successful in none of the PDX-induced granulomas. All culture-positive granulomas had large numbers of AFB within the necrosis but variable and low numbers of AFB in the inflammatory infiltrate. 

The comparison between the detection of AFB by Ziehl–Neelsen staining and of mycobacterial antigen by immunohistochemistry (IHC) revealed no differences in strongly positive sections, notably in the necrosis (Table 3). IHC-based detection was more sensitive in the inflammatory infiltrates of VPM1002-, PDX-, and NUOG-induced granulomas. Only in one granuloma were AFB but no mycobacterial antigens present. The combination of both labeling methods allows for the detection of clearly defined AFB with the morphology of bacilli (Figure 16) and has the advantage of the higher sensitivity of antigen detection (Table 3).

## 3. Discussion

Granulomas are the hallmark of tuberculosis. Reproducible granuloma models which closely reflect human disease-associated lesions, e.g., caseous granulomas, are important, because they allow for insights into host–pathogen interactions. All goats (except two that had received PDX) developed caseous granulomas at the injection site in response to the BCG vaccines, while no lesions were seen after the injection of PBS. The high rate of goats with vaccination granulomas in our trial may be due to the close clinical monitoring of the injection site, which allowed for the collection of even small lesions at necropsy [22,26]. Another factor could be the time of collection, since healing can occur with time [24,26] and was seen in some of the goats that had received PDX. 

Subcutaneous granulomas have several advantages as a model system. Histological examination confirmed the characteristic organization of tuberculous granulomas with central, partly calcified necrosis surrounded by an inflammatory cell infiltrate and a fibrous capsule. This is in accordance with the description of BCG-induced granulomas in goats [26] and tuberculous granulomas in goats [4,26], cattle [16] and humans [15]. Following the commonly used classification scheme for bovine tuberculosis [42], all granulomas were type 3 granulomas. 

The uniformity in the morphology and organization of the subcutaneous granulomas in this trial differs from the heterogeneity observed in natural and experimental infections, where granulomas are most frequent in the lung and draining lymph nodes [48,49,50]. The application of a defined dose of mycobacteria at a defined time into loose connective tissue containing few immune cells most likely reduced confounding factors contributing to granuloma heterogeneity, as changes in the morphology of tuberculous granulomas were seen in longitudinal studies [51]. This uniformity facilitated the comparison between granulomas induced by different vaccines. Variations between individual goats are most likely related to the fact that goats are an outbred, genetically heterogenous species. In this respect, they are more comparable to humans than laboratory rodents [52]. Differences between vaccine groups sometimes only revealed trends but did not reach levels of significance because of these variations and the low numbers of animals per group.

Another advantage of the subcutaneous granuloma model is that it places less pain, suffering and distress on the animals, since, in this experiment, only transient local inflammation occurred, and no systemic clinical signs were induced [27]. Thus, subcutaneous vaccine granulomas in goats are a highly reproducible model for tuberculous granulomas. The complex organization of these dynamic structures reflects multiple interactions between mycobacteria, their target cells as well as non-specific and specific components of the host immune system [12,15,16,53,54].

A detailed investigation of the structural components of granulomas induced by different vaccines revealed differences that reflect the changes in the in vivo behavior of the recombinant BCG derivates. At the macroscopic level, the granulomas induced by the recombinant BCGs, especially VPM1002, were larger compared to those induced by BCG. An increase in granuloma size has been primarily linked to an increase in central necrosis [15,47], but there was also an increase in the inflammatory infiltrate in the VPM1002- and PDX-induced granulomas which was indicative of a stronger immune response. Interestingly, the inflammatory cell infiltrate was particularly small in NUOG-induced granulomas. *NuoG* was initially identified as a virulence gene of *M. tuberculosis,* encoding the *nuoG* subunit of the type I NADH-dehydrogenase, which inhibits apoptosis [55]. Apoptosis of infected cells is considered beneficial for the host because it causes less tissue damage than necrosis and promotes increased antigen presentation [32,56]. The deletion of *nuoG* in VPM1002 only slightly increased apoptosis compared to VPM1002 alone but unexpectedly increased pathogen-targeted autophagy (xenophagy) in THP1 macrophages [41]. In murine lymph nodes, apoptosis was significantly increased at a later stage (day 14) after NUOG vaccination compared to vaccination with the parental VPM1002 strain. Overall, vaccination with NUOG resulted in the improved clearance of *M. tuberculosis* from the lung of mice and enhanced immune responses compared to VPM1002 [41].

In goats, central necrosis with calcifications and cellular debris was comparable in granulomas irrespective of the vaccine type used. Numerous AFB and large amounts of mycobacterial antigens were located in most granulomas, even 4 months after the application of the mycobacteria. Mycobacteria have been reported to use the caseum as an immunological niche for survival [57,58,59]. We could confirm by immunohistochemistry that this immunological niche was not accessed by blood vessels and inflammatory cells, whereas the surrounding inflammatory infiltrate was well vascularized. The distribution of mycobacteria was not diffused throughout the necrosis but multifocal, with higher numbers associated with nuclear fragments and calcification. A comparable distribution was reported for persisting *M. tuberculosis* in guinea pigs with an extracellular location in biofilm-like structures consisting of DNA and disintegrated neutrophils in a hypoxic and iron-rich environment with dystrophic calcification [60]. Mycobacteria were not cultured from most granulomas induced by VPM1002 and NUOG and from any granuloma induced by PDX, indicating that they were either not viable or viable but not culturable. In at least some of the vaccine-induced granulomas, the mycobacteria in the caseum were viable, since the cultural isolation of BCG was possible in granulomas where bacteria were only detected within the necrosis.

Lipid droplets, as detected by appropriate staining, were present in the necrotic centers of granulomas induced by BCG vaccination, but they were lacking in the necrotic centers of granulomas induced by the recombinant vaccines. Lipids are considered a source of nutrients for the mycobacteria in the caseum [58]. A lack of lipids in the area of central necrosis may impair long-term survival of the recombinant BCGs in the caseum and thus contribute to the safety of these vaccines. The lipid droplets in the caseum originate from lipid droplets in epithelioid cells and MNGCs termed foam cells because of their vesiculated cytoplasm in histological preparations [57,61]. Foam cells are present at the interface between the necrosis and the inflammatory infiltrate, and lipid droplets enter the necrotic area via the decaying cells. The formation of foam cells correlates with the pathogen-mediated dysregulation of host cell lipid metabolism [58]. In an in vitro human granuloma model, the differentiation of macrophages into foam cells was induced by oxygenated mycolic acids from *M. tuberculosis* [57]. Increased lipid metabolism was also demonstrated in cells surrounding the caseum in human tuberculous granulomas [62] but is not consistently seen in bovine granulomas [16]. Not all mycobacteria are able to modify the metabolism of macrophages: *M. tuberculosis* and *M. avium*, but not *M. smegmatis*, induce foam cells [57]. Our data indicate that the genetic modifications of the recombinant BCG vaccines may have abrogated their ability to dysregulate the host lipid metabolism.

Mycobacteria residing in the caseum in a metabolically downregulated state use cholesterols in addition to lipids as nutritional provisions [58]. Cholesterol originates predominantly from the cellular membranes of decaying cells at the edge of the area of necrosis—mainly, epithelioid cells, MNGCs and neutrophils [61]. In VPM1002-induced granulomas, the number of neutrophils at the edge of the necrotic area was particularly high. Neutrophils participate in the innate response to mycobacteria by direct interaction with the mycobacteria, e.g., phagocytosis, the release of cytokines and chemokines, degranulation and the formation of neutrophil extracellular traps, and indirectly by networking with other inflammatory cells, e.g., macrophages, dendritic cells, platelets and lymphocytes [63]. It remains unclear if these effects are beneficial or detrimental for the host [63,64]. The strong influx of neutrophils into VPM1002-induced granulomas may result in an increased loss of these cells to the caseum, contributing to the increased size of the central necrotic area in VPM1002-induced granulomas.

The overall organization of the inflammatory infiltrate of granulomas varied from a stratified organization with epithelioid cells almost exclusively in the central region and lymphocytes in the periphery to an even distribution of epithelioid cells and lymphocytes. This was not associated with the different vaccine types. In the zebrafish-*M. marinum* model, the stratified organization of granulomas was dependent on the reprogramming of macrophages to express epithelial molecules, e.g., E-cadherin, and adherence junctions [59]. This might reduce the access of immune cells to mycobacteria and mycobacteria-infected cells and thus protect the mycobacteria from an immune response [59]. The cause for the differential organization between individual animals, irrespective of the vaccine applied and even within individual granulomas, remains unresolved. 

A major cell type of the inflammatory cell infiltrate is the epithelioid cell. Epithelioid cells are macrophages activated either by infection with mycobacteria or by being recruited by inflammatory mediators released from infected macrophages. The low number of epithelioid cells staining positive for AFB or mycobacterial antigen observed in the vaccine-induced granulomas confirms that only a fraction of these cells is infected [65]. There was no difference in the number of epithelioid cells between the vaccine groups, but they were rather large and had extensive cytoplasm in NUOG-induced granulomas. Epithelioid cells are the initial targets of mycobacteria. The complex processing of the mycobacteria in these cells, e.g., the maturation of the phagosome, phagosome–lysosome fusion, phagosome acidification and the ability of mycobacteria to disrupt the phagosome, leading to cytosolic access, influences the outcome of infection [65,66,67]. In particular, the ability to translocate mycobacterial proteins or DNA to the cytosol differs between mycobacteria. While BCG remains inside the phagosome [68], because it does not have a functional type VII secretion system [69], the recombinant BCG-vaccine VPM1002 and its derivatives were designed to allow for cytosolic access via listeriolysin [28,29,30]. The phenotypic outcome is similar to that of other mycobacteria, e.g., *M. tuberculosis* and *M. bovis*, which use a type VII secretion system to damage and rupture the phagosomal membrane and access the cytosol. Mycobacterial components are recognized as a pathogen-associated molecular pattern by Absent in Melanoma 2 (AIM2) and trigger various signaling pathways that result in cytokine production, inflammasome activation, autophagy and apoptosis [30,32,33,70,71]. Further ultrastructural studies should elucidate whether the unusual morphology of epithelioid cells in NUOG-induced granulomas is associated with distinct intracellular alterations induced by NUOG.

Another cell type in the inflammatory infiltrate originating from macrophages are MNGCs, which were present in markedly lower numbers in VPM1002-, PDX- and NUOG-induced granulomas compared to those induced by BCG. MNGCs develop in response to chronic antigenic stimuli either by the fusion of pro-inflammatory macrophages or by multinucleation due to mitotic defects [72,73,74]. Both mycobacterial factors, e.g., cell wall components and secreted proteins, and host factors, e.g., mediators secreted as extracellular vesicles by infected macrophages, are required for MNGC formation [75]. It has been reported that they are triggered by virulent—but not by avirulent—mycobacteria [76]. Thus, the reduced number of MNGCs in granulomas induced by the recombinant BCGs suggests their virulence is reduced compared to BCG, as seen in the mouse model [28]. MNGCs display reduced phagocytic activity and cytokine patterns distinct from epithelioid cells [76,77]. Therefore, the containment of mycobacteria has been considered to be the main function of MNGCs [59]. The reduced number of MNGCs in granulomas induced by the recombinant BCGs may promote the contact of mycobacteria with inflammatory cells and a stronger induction of immune responses. 

Epithelioid cells and MNGCs, if present, were surrounded by MHC II^+^ cytoplasmic extensions of DCs in all granulomas. The presence of DCs even 4 months after the application of the vaccines indicates a continued immune stimulation. DCs can become infected with *M. bovis* and BCG and provide an environment where mycobacteria survive and replicate [78]. DCs may also acquire fragments or antigens of mycobacteria, especially if primary infected cells undergo apoptosis. The modified subcellular processing of the recombinant BCGs in epithelioid cells may promote increased antigen presentation by DCs [32,35,56]. Infected DCs or DCs carrying mycobacterial antigens migrate to regional lymph nodes, where they present antigens directly or via cross presentation to CD4^+^ and CD8^+^ T cells. Activated T cells recirculate and promote granuloma formation [79]. In response to mycobacterial infection, DCs secrete cytokines and change their surface molecule expression patterns. The observation that the expression of cytokines was markedly increased in *M. bovis*-infected DCs compared to BCG-infected DCs might indicate differences in intracellular processing [78]. Since mycobacteria reside in membrane-bound vacuoles in DCs [80], LLO might affect this membrane in a similar way as the phagosome membrane in macrophages and epithelioid cells, modify the intracellular processing of recombinant BCG and contribute to the improved immune reaction [28,30,35,81]. 

The modulation of subcellular processing in infected cells by the recombinant vaccines was reflected by the lymphocyte subsets infiltrating the granulomas. There were significantly higher numbers of CD8^+^ T cells in all granulomas induced by the recombinant BCGs as compared to BCG-induced granulomas. This is particularly interesting, as VPM1002 was originally designed with the intent of eliciting improved CD8^+^ T cell responses by increasing apoptosis [28], but increased CD4 T^+^ cell responses rather than increased CD8^+^ T cell responses were observed in the mouse model [36]. An increase in CD8^+^ T cells in the peripheral blood was also seen in human newborns vaccinated with VPM1002 [38]. CD8^+^ T cells contribute to the elimination of mycobacteria by the production of pro-inflammatory cytokines and by releasing the content of cytotoxic granules, which directly exert mycobactericidal effects [79,82,83,84]. There were no significant changes between the BCG- or recombinant BCG-vaccinated groups in terms of the number of CD4^+^ T cells, which are considered essential for the maintenance of granulomas [16,79]. However, changes in the functional subtypes of CD4^+^ T cells, e.g., effector memory and central memory CD4^+^ T cells, and polarization to Th1 and Th17 cells, which are important for protection, were not investigated in the goats [29,35,36,41]. γδ T cells were present in all granulomas in low numbers. The increase in the number of these cells in the lungs of mice after vaccination with VPM1002 in comparison to BCG was not observed in our subcutaneous granuloma model. Since γδ T cells are predominantly associated with mucosal barriers, where they are important as the first line of defense against infection [85,86], changes are more likely to occur in the lung. Another reason for the low numbers in our model might be the age (4 months) of the granulomas, because γδ T lymphocytes are known to be important early in the development of granulomas and to then decline in number [87]. In the vaccine-induced granulomas, γδ T cells were evenly distributed throughout the inflammatory infiltrate. This differs from granulomas in patients with TB, where γδ T cells are present in the necrotic zone of granulomas in lymph nodes [88] or as rings around the necrotic zone in pulmonary granulomas [53]. We also stained B cells and found that their numbers were significantly reduced in VPM1002- and NUOG-induced granulomas. They were not evenly distributed but formed follicle-like aggregates in all granulomas, except those induced by NUOG. This so-called tertiary lymphoid tissue is assumed to play a role in the maintenance of intact granulomas [89]. 

Overall, the data presented here argue in favor of a more balanced immune response with a stronger activation of CD8^+^ T cells in goats vaccinated with the recombinant BCG-derivatives, as intended by the targeted design and construction of these vaccine strains. 

## 4. Materials and Methods

### 4.1. Animals

Thirty conventionally raised male goats of the German Improved White breed were included in this study. The animals were transfered at 8 weeks of age, with an average weight of 25 kg, from a conventional farm without a history of tuberculosis to the animal facility of the Friedrich-Loeffler-Institut in Jena. The goats had been castrated and vaccinated against *Clostridium* spp., *Mannheimia haemolytica* and *Pasteurella trehalosi* (Heptavac^®^ p plus, Intervet Deutschland GmbH, Unterschleißheim, Germany) in their herd of origin.

Upon arrival, the health status was controlled by the microbiological and parasitological examination of nasal swabs and fecal samples [27]. Goats were housed in groups in separate air-conditioned loose-boxes with natural daylight and straw bedding. Throughout the entire study, the animals were reared under standardized conditions (room climate: 19 °C ± 2 °C, humidity: 50% ± 20%, natural light) and in accordance with international guidelines for animal welfare. They were fed hay and age-adjusted amounts of concentrated feed. Water was provided ad libitum. 

The actual health status of the goats was determined by daily clinical examination. Most of the animals had spontaneous coughing and mild nasal or conjunctival discharge at arrival. For this, they received antibiotic treatment for 5 days with Enrofloxacin (Baytril^®^, Bayer, Leverkusen, Germany), four weeks before experimental vaccination. They were treated with Toltrazuril (Baycox^®^ 5%, Bayer) to limit coccidial infection. 

This study was carried out in strict accordance with the European and National Law for the Care and Use of Animals. The protocol was reviewed by the Committee on the Ethics of Animal Experiments of the State of Thuringia, Germany and approved by the competent authority, the Animal Health and Welfare Unit of the Thuringian State Office for Consumer Protection (Permit Number: 22-2684-04-04-001/16). All experiments were performed in a containment of biosafety level 2 and gentech level 1 under supervision of the authorized institutional Agent for Animal Protection. During the entire study, every effort was made to minimize suffering. 

### 4.2. Vaccination 

At 5 months of age, six goats each were vaccinated with one of the vaccine candidates—VPM1002, PDX, NUOG, BCG SSI—or with phosphate buffered saline (PBS) as a mock-treatment. The candidate vaccine VPM1002 and its derivatives PDX and NUOG, as well as BCG strain SSI, were provided by the Max Planck Institute for Infection Biology (MPIIB, Berlin, Germany) and delivered as cryo-conserved aliquots. The original stock of BCG SSI 1331 Danish ATCC 357533 was obtained from American Type Culture Collection. The mycobacteria were washed three times with PBS and reconstituted in PBS with an intended bacterial count of 5 × 10^5^ CFU mycobacteria per dose. Vaccines were administered subcutaneously behind the left scapula at a volume of 500 µL. Prior to vaccination, the area was shaved and disinfected. The actual total doses, as determined by re-titration, were 7.3 ± 4.7 × 10^7^, 3.7 ± 1.5 × 10^7^, 13.4 ± 5.8 × 10^7^ and 1.6 ± 1.1 × 10^7^ CFU (mean + SD of three independent cultures) per goat for VPM1002, PDX, NUOG and BCG, respectively. 

### 4.3. Clinical Examination and Findings after Vaccination

A comprehensive clinical examination of the animals was carried out daily and documented using a scoring system. Post-vaccination, the examination included the injection sites of vaccines, where the size of lesions, redness, pain, swelling, local temperature and necrosis were recorded [27]. All animals vaccinated with BCG, VPM1002, NUOG or PDX initially developed edematous swelling at the injection site and later developed solid subcutaneous nodules. Approximately 1 month after vaccination, one to three goats in all groups developed small ulcerations in the overlaying skin, with the draining of exudate observed in NUOG- and BCG-vaccinated goats. After 2 to 3 days, ulcerations were sealed with a scab, which remained for up to 3 months. Goats vaccinated with NUOG and VPM1002 had larger lesions than goats vaccinated with PDX or BCG (for scoring, see [27]). 

### 4.4. Necropsy, Gross Pathology and Tissue Samples 

Four months after vaccination, the goats were euthanized by an intravenous injection of 100 mg/kg Pentobarbital-sodium (Release 500 mg/mL^®^, WDT, Garbsen, Germany) following sedation by an intramuscular injection of 0.25 mg/kg Xylazin (Rompun^®^ 2%, Provet AG, Lyssach, Switzerland). Complete necropsies with the macroscopic assessment and histologic examination of representative organs and tissues were performed [27]. For the sampling of the vaccination site, a square field of skin with an edge length of about 5 cm above the subcutaneous nodule was deeply incised and removed together with the nodule. The surrounding connective tissue and subcutaneous muscle were dissected from the nodule, the size of the nodule was measured and its consistency was palpated. Then, it was cut with a sterile scalpel to assess the cut surface and contents. Each nodule was divided in three aliquots: one was immersed in 4% neutral buffered fomalin for histologic examination, one was snap frozen at −70 °C for immunohistochemistry and one was collected under sterile conditions for the cultural isolation of mycobacteria. 

### 4.5. Histology

Tissues for histologic evaluation were embedded in paraffin (FFPE). FFPE sections were stained with hemalaun and eosin (HE) for overall morphologic assessment. Azan, von Kossa, Sudan III and Ziehl Neelsen (ZN) staining were performed for further differentiation. 

Azan staining, which was also carried out on formalin-fixed frozen sections, stains collagen fibers. The direction, size and staining intensity of fibers allowed for the distinction from pre-existing subcutaneous connective tissue, precise measurments of the width of inflammatory infiltrates and the placing of reference areas for cell counts. The width of inflammatory infiltrates was measured at eight equidistant sites per granuloma. If only a part of the granuloma was present, the number of sites measured was reduced accordingly. 

Von Kossa staining was used to visualize calcium ions to characterize and quantify calcification of the caseous necrosis. Semiquantitative scoring of mild (+, <10% of necrosis calcified), moderate (++, 10–50% of necrosis calcified) and severe (+++, >50% of necrosis calcified) necrosis was applied. 

Sudan III staining was performed on formalin-fixed frozen sections to visualize lipids. The number of cells with lipid droplets along the edge of the caseous necrosis was scored as a single or few cells with a variable number of lipid droplets per granuloma (+), a few cells with lipid droplets in each 40x-high power field (hpf, ++) or numerous cells with lipid droplets in each hpf (400×) (+++). 

Acid-fast bacteria (AFB) were staind by ZN. The number of AFB was determined within caseous necrosis and inflammatory infiltrate as few (+, <10% of cells with few AFB and/or few/focal AFB in the necrosis), many (++, 10–50% of cells with few AFB or 10–30% of cells with many AFB and/or multifocal small groups of AFB in the necrosis) and numerous (+++, >50% of cells with few AFB or >30% of cells with many AFB and/or numerous, multifocal to diffuse AFB in the necrosis).

### 4.6. Immunohistochemistry 

Monoclonal antibodies to CD4, CD8, δ chain, CD79α, CD68, MHC class II as well as polyclonal antisera to factor VIII and mycobacterial antigen were used to detect helper T cells, cytotoxic T cells, γδ T cells, B cells, plasma cells, macrophages including epithelioid cells, MNGC, DCs, endothelial cells and mycobacteria/BCG in frozen or paraffin sections (Table S1). 

Frozen sections were air dried, fixed in acetone and rehydrated. Endogenous peroxidase was inhibited by incubation with 0.06% phenylhydrazine in PBS at 37 °C for 40 min. FFPE sections were deparaffinized and rehydrated. They were treated with proteinase K for the retrieval of factor VIII or with trypsin for the retrieval of mycobacterial antigen. Endogenous peroxidase was inhibited with 0.3% H_2_O_2_ in methanol for 25 min at room temperature. Non-specific binding was blocked in frozen and paraffin sections by incubation with inactivated serum of the species from which the secondary antibody originated. After incubation with the respective primary antibodies, indirect immune peroxidase or the avidin-biotin-complex method were performed (Table S1). Diaminobenzidine was used as the chromogen and was intensified with 0.01% osmic acid. Sections were counterstained with 2% methylene green and cover slipped with Kaiser’s glycerin gelatin. Positive controls (sections containing the specific cell types/mycobacteria) and negative controls (first antibodies replaced by an antibody against an unrelated antigen) were included in each reaction.

For quantitative assessment, an Axio Imager 2 microscope with an Axiocam 305 color digital camera and the software Zen pro (Carl Zeiss, Oberkochen, Germany) were used. The number of CD4^+^ T cells, CD8^+^ T cells, γδ T cells, B cells, plasma cells, macrophages/epithelioid cells and MNGCs was counted at five sites per granuloma (Figure S1). The first site was randomly selected, and the next four sites followed clockwise around the granuloma at equal distances. If the section of a granuloma was incomplete, the first and fifth sites were selected, and the remaining three sites were evenly distributed. At each site, the cells were counted in three 100 µm × 100 µm reference areas (RAs) which were positioned as follows: centrally bordering the caseous necrosis, at the periphery bordering the fibrous capsule and between these positions (Figure S1). RAs that were 200 mm × 100 µm in size were defined for MNGCs (Figure S1).

### 4.7. Bacterial Culture 

From each granuloma, 1 g of tissue containing both caseous necrosis and solid wall was placed into 10 mL PBS and homogenized for six minutes at room temperature using a stomacher. A total of 10 mL of NALC-NaOH solution (containing natrium-citrat-dihydrat 2.9% and N-acetyl-L-cystein 0.5%) were added, and the sample was agitated for 25 min at 300 rpm on a shaker. After the addition of 20 mL PBS, the sample was vortexed and centrifuged for 20 min at 3800× *g*. The supernatant was discarded, 10 mL PBS was added and the sample was centrifuged as before. The supernatant was discarded, and the pellet was resuspended in 1 mL PBS and homogenized thoroughly by vortexing. From each resuspended pellet, 200 µL were transferred to one slant of Löwenstein–Jensen medium with Polymyxin B, Amphotericin B, Carbenicillin, Trimethoprim (PACT) and Glycerin and to two slants of Coletsos medium with PACT (both Artelt-Enclit, Borna, Germany). The tubes were incubated for 1 week in a horizontal position and then incubated afterwards in an upright position for up to 12 weeks. Cultures were checked every two weeks for colony growth. When visible colonies appeared, the presence of *M. bovis* was confirmed by real-time PCR targeting IS*1081* and by endpoint PCR targeting RD4 [90,91]. In-house primers were used for the identification of VPM1002 and its derivatives [27].

### 4.8. Statistical Analysis 

A statistical analysis was performed for the measurements of the granulomas’ sizes, the width of the inflammatory cell infiltrate and the cell counts using SPSS (IBM SPSS Statistics for Windows). The Mann–Whitney-U test with a significance level at 0.05 (*p* ≤ 0.05) was used for the pairwise comparison between the different vaccination groups. All data were plotted as boxplots showing the median (horizontal line), the interquartile range (box), and the minimum and maximum (whiskers) using GraphPad Prism (version 9, Dotmatics, San Diego, CA, USA). All data beyond 1.5 times the interquartile range are depicted as outliers (dots).

## 5. Conclusions

All components described in human TB granulomas were present in the vaccine-induced subcutaneous granulomas of goats. The immune system of the goats recognized and reacted distinctively to the genetic modifications of the recombinant BCG vaccines. These subcutaneous granulomas represent an additional model for a better understanding of the cellular composition and function of granulomas and for uncovering differences in immune responses to novel vaccines. Its advantage compared to granulomas in zebrafish is that the immune system of goats more closely resembles that of humans. As such, it may also be useful as a model for characterizing the nature of advanced-stage human TB vaccine candidates. Furthermore, this in vivo model reflects not only the local reaction but also the responses induced in regional lymph nodes. The fine tuning of the model in goats may involve the sequential collection of granulomas, the identification of cellular subtypes and cytokines by in situ hybridization, as described for cattle [92], or global analyses such as gene expression and proteomic profiling at a tissue level [93]. Studies testing the immunogenicity and protection of the different recombinant vaccines in a challenge infection in goats will follow. This will reveal if the reactions to the vaccines in the subcutaneous tissues of goats may serve as an indicator of handling an infection with virulent *M. bovis*. 

## 6. Patents

S.H.E.K and L.G. are co-holders of a patent on the tuberculosis vaccine, VPM1002, licensed to Vakzine Projekt Management GmbH, Hannover, Germany and Serum Institute India Pvt. Ltd., Pune, India. 

## Figures and Tables

**Table 1:** Calcification in the central necrosis of caseous granulomas of goats (n = 6 per vaccine group) after subcutaneous injection of BCG, VPM1002, PDX or NUOG.

BCG		VPM1002		PDX		NUOG	
Distrib.	Amount	Distrib.	Amount	Distrib.	Amount	Distrib.	Amount
S	+++	S	++	S	++	D	+++
D	+++	S	++	---	---	D	+++
D	++	D	+++	---	---	D	+++
D	++	D	++	D	+	D	+++
D	+++	S	++	D	+	D	+++
D	++	S	++	D	+	D	+++

S—solid, coarse; D—dusty, granular; + <10% of necrosis calcified; ++ 10–50% of necrosis calcified; +++ >50% of necrosis calcified; --- no central necrosis.

**Table 2:** Lipid droplets in foam cells in caseous granulomas of goats (n = 6 per vaccine group) after injection of BCG, VPM1002, PDX or NUOG.

Vaccine	Overall Amount in Granulomas of Individual Goats	Distribution of Lipid Droplets	Size of Lipid Droplets
BCG	+++	in epithelioid cells and MNGC, esp. close to necrosis, less in periphery, in central necrosis	small lipid droplets, numerous lipid droplets per cell, sometimes confluent
	+++		
	+++		
	+++		
	+++		
	+++		
VPM1002	+	in epithelioid cells	pleomorphic, few per cell
	+		
	+		
	+		
	+		
	+		
PDX	+	in epithelioid cells	pleomorphic, few per cell
	---		
	---		
	-		
	-		
	-		
NUOG	++	in epithelioid cells	highly variable in size and shape, some large
	++		
	---		
	++	in epithelioid cells	highly variable in size and shape, some large
	++		
	++		

+ lipid droplets in single/a few cells, ++ lipid droplets in many cells, +++ lipid droplets in numerous cells, - no lipid droplets, --- no caseous necrosis/inflammatory infiltrate available for evaluation.

**Table 3:** AFB, mycobacterial antigen and cultural isolation of BCG in caseous granulomas of goats (n = 6 per vaccine group) after subcutaneous injection with BCG, VPM1002, PDX or NUOG.

Vaccine	In Caseous Necrosis		In Inflammatory Infiltrate		Cultural Isolation
	AFB	AG	AFB	AG	
BCG	+++	+++	+	+	c
	+	+	-	-	c
	+++	+++	+	+	-
	+++	+++	-	-	c
	+	++	+	-	-
	+	+	-	-	-
VPM1002	+++	+++	+++	+++	-
	++	++	++	++	-
	+++	+++	-	-	-
	++	+++	+	+++	c
	+++	+++	-	+	c
	+	++	-	++	-
PDX	-	+	-	+	-
	++	+++	-	-	-
	-	-	-	-	-
	++	---	++	+++	-
NUOG	+	+	-	-	-
	-	-	-	-	-
	-	+	+	++	-
	+++	+++	+	+	c
	+++	+++	-	-	-
	++	+++	+	+++	-

c culture positive; AFB acid-fast bacilli; AG mycobacterial antigens; - no AFB/no antigen/culture-negative; + few, <10% of cells with few AFB and/or few/focal AFB in the necrosis; ++ many, 10–50% of cells with few AFB or 10–30% of cells with many AFB and/or multifocal small groups of AFB in the necrosis; +++ numerous, >50% of cells with few AFB or >30% of cells with many AFB and/or numerous, multifocal to diffuse AFB in the necrosis; --- no caseous necrosis/inflammatory infiltrate available for evaluation.

## Data Availability

The data presented in this study are available on request from the corresponding author.

## Supplementary Materials

The supporting information can be downloaded at: https://www.mdpi.com/article/10.3390/ijms231910992/s1.
